# Evaluation of Spontaneous Pain in a Monosodium Iodoacetate-Induced Mouse Model of Osteoarthritis Using the Mouse Grimace Scale

**DOI:** 10.3390/biomedicines14092114

**Published:** 2026-09-19

**Authors:** Mohammed Alnoud, Jose Rios, Megan W. Szobody, Ayesha A. Usman, Alma Rodriguez, Thao-April Nguyen, Seryna Ayala, Ayaan S. Quraishi, Massimo Salinas, Khalid Benamar

**Affiliations:** Department of Neuro-Behavioral Health, Institute of Neuroscience, School of Medicine, University of Texas Rio Grande Valley, McAllen, TX 78504, USA

**Keywords:** Osteoarthritis, pain, ongoing pain, Mouse Grimace Scale, monosodium iodoacetate

## Abstract

Animal models are critical for elucidating disease mechanisms and pathological outcomes, and for evaluating therapeutic interventions. Osteoarthritis (OA) is a leading cause of chronic pain and disability globally. Clinically, individuals with OA experience both sensory and ongoing pain. However, ongoing pain, as assessed by the Mouse Grimace Scale (MGS), has not yet been evaluated in animal models of OA. This study aims to address this significant knowledge gap. We induced OA by intra-articular injection of monosodium iodoacetate (MIA), while control mice received saline injections. We assessed pain behaviors using the MGS on days 1, 3, 7, and 14 after MIA administration. MIA-treated mice exhibited significantly higher MGS scores than saline-treated controls throughout the study period, indicating ongoing pain-related behavior in the MIA OA model. Furthermore, longitudinal studies showed that this pain is chronic, as seen in people with OA. These findings support the usefulness of the MGS test in the MIA model for studying ongoing pain.

## 1. Introduction

Osteoarthritis (OA) is the most common form of arthritis and a leading cause of chronic pain and disability worldwide [1,2]. OA is a whole-joint disease that involves all joint tissues, including meniscal degeneration and inflammation of the infrapatellar fat pad [3,4,5,6]. OA-related pain remains the most prominent and disabling symptom, determining patients’ functional limitations and quality of life [7,8]. Established risk factors for OA include older age, female sex, overweight and obesity, genetic predisposition, and previous major joint injury [9].

Animal models have become essential tools for investigating OA pathogenesis, identifying pain mechanisms, and evaluating potential therapeutic interventions [7,8]. Several experimental approaches have been developed to study OA [9,10]. Among these models, chemically induced OA models have gained widespread use due to their reproducibility [11,]. Among chemically induced OA models, the monosodium iodoacetate (MIA) model is one of the most extensively characterized and widely used preclinical models for investigating OA-associated pain [12,13,14]. Intra-articular administration of MIA inhibits glyceraldehyde-3-phosphate dehydrogenase activity in chondrocytes, disrupting cellular glycolysis, leading to chondrocyte death, progressive cartilage degeneration, subchondral bone remodeling, and synovial inflammation [15]. These pathological changes are accompanied by pain.

Translational, validated animal models are essential for understanding disease mechanisms and pathological outcomes and for testing therapeutic interventions. For chronic pain, animal models that mimic sensory and ongoing pain are more suitable and translational because they reflect aspects of chronic OA pain in humans.

The MGS has emerged as a reliable, noninvasive tool for assessing pain-related spontaneous behavior in laboratory animals by evaluating pain-related facial expressions [10,11].

So far, this test has not assessed ongoing pain in MIA or any OA rodent models. This study aims to address this significant knowledge gap by characterizing ongoing pain-related behavior in MIA model using MGS.

## 2. Materials and Methods

### 2.1. Animals

Adult female C57BL/6 mice (30–35 g) were obtained from Jackson Laboratory (Bar Harbor, ME, USA). Animals were group-housed (4–5 mice per cage) under controlled environmental conditions (22 ± 2 °C; 12 h light/dark cycle) with free access to standard laboratory chow and water. Mice were acclimated to the animal facility for at least one week before experimentation. All experimental procedures were approved by the Institutional Animal Care and Use Committee (IACUC) at the University of Texas Rio Grande Valley and were conducted in accordance with the National Institutes of Health Guide for the Care and Use of Laboratory Animals. We conducted all experiments in a blind, random manner.

### 2.2. Induction of Osteoarthritis

Experimental OA was induced by a single intra-articular injection of monosodium iodoacetate (MIA) (1 mg in 10 µL of sterile saline) into the right knee joint under isoflurane anesthesia (3–4% for induction and 1–2% for maintenance). Control mice received an equivalent volume (10 µL) of sterile saline via the same procedure. After injection, we monitored all mice until full recovery, then returned them to their home cages.

### 2.3. Pain-like Behavioral Measures

#### Mouse Grimace Scale (MGS)

Manual scoring. We assessed pain-related spontaneous behavior using the MGS as previously described [10]. We placed mice individually in transparent Plexiglas chambers and allowed them to acclimate before video recording. We recorded facial expressions for 5 min using digital cameras positioned to obtain clear frontal and lateral views of each animal. We manually analyzed and quantified the videos based on four MGS facial action units: orbital tightening, nose bulge, ear position, and whisker change. Each facial action unit was scored on a three-point scale from 0 to 2, where 0 indicated the absence of the facial feature, 1 indicated a moderate or equivocal presence, and 2 indicated an obvious presence. We used the average MGS score for each animal in statistical analysis.

PainFace. We analyzed recorded videos using the cloud-based PainFace platform [12], an automated, machine-learning-based software developed to standardize and scale mouse grimace analyses. PainFace automatically detects four MGS facial action units: orbital tightening, nose bulge, ear position, and whisker change. Each facial action unit is assigned a score based on the degree of the facial expression, and the MGS score for each analyzed frame is calculated by summing the scores of the four facial action units, resulting in a total score ranging from 0 to 2. PainFace generates MGS scores for the analyzed video frames, and we used the average MGS score for each animal for statistical analysis.

### 2.4. Statistical Analysis

Statistical analyses were performed in GraphPad Prism v11 (GraphPad Software; San Diego, CA, USA) using Two- Way ANOVA with Bonferroni’s Post Hoc Tests. A value of *p* < 0.05 was considered statistically significant.

## 3. Results

### 3.1. Experimental Design

OA was induced by intra-articular (MIA) injections, whereas control mice received intra-articular saline injections. We assessed pain-related behaviors on days 1, 3, 7, and 14 after injection.

### 3.2. MIA Induces Pain-Related Spontaneous Behaviors Assessed by Manual MGS

**Figure 1:**
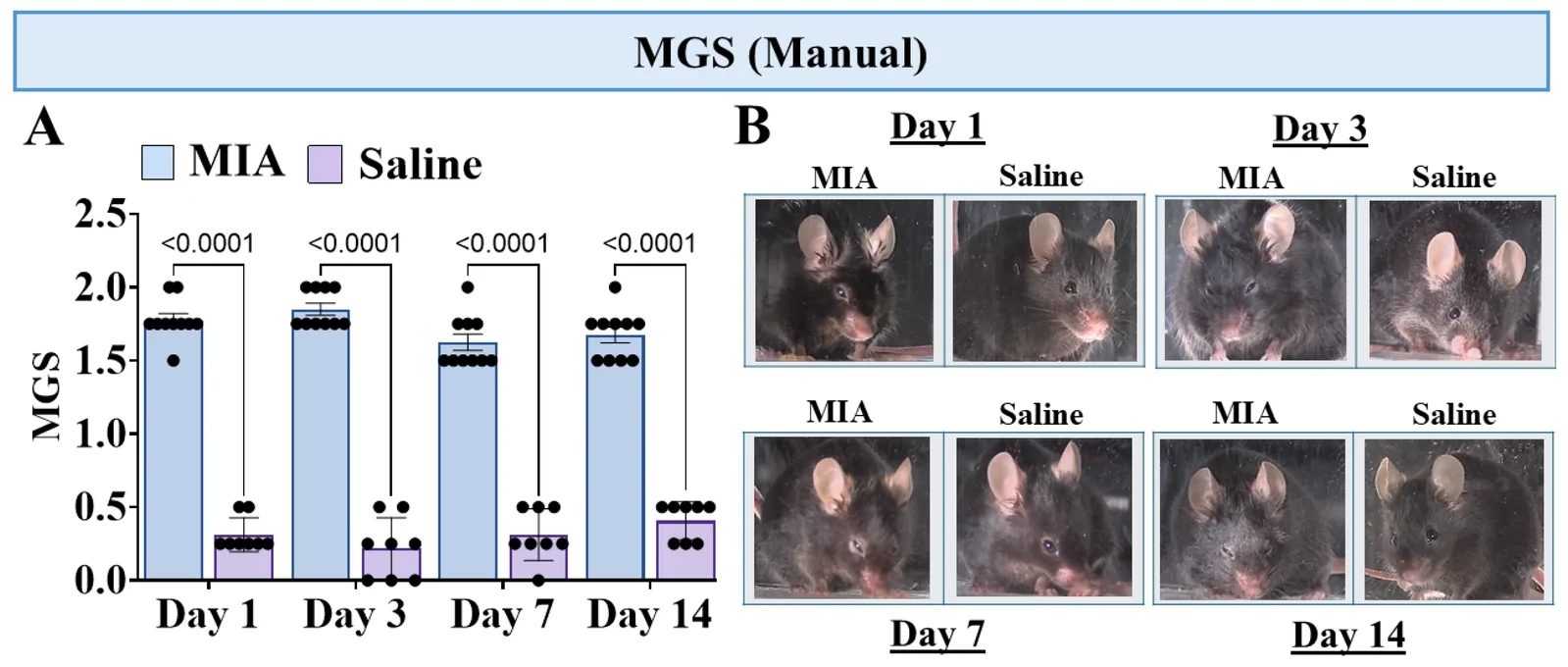
MIA induces pain-related spontaneous behaviors after intra-articular injection using manual MGS. Representative facial images of MIA- and saline-treated mice on days 1, 3, 7, and 14 (**A**). Representative facial images of MGS scores on days 1, 3, 7, and 14 (**B**). Data are presented as mean ± SEM. *n* = 8–10 mice per group.

After intra-articular injection of MIA or saline, we assessed pain-related spontaneous behaviors on days 1, 3, 7, and 14 using the MGS. Manual MGS scoring revealed significantly higher facial pain scores in MIA-treated mice than in saline-treated mice on days 1, 3, 7, and 14 (Figure 1A and Two-Way ANOVA). Representative facial images showed distinct pain-associated facial expressions in MIA-treated mice compared with saline-treated controls on days 1, 3, 7, and 14 (Figure 1B).

### 3.3. MIA Induces Pain-Related Spontaneous Behaviors Assessed by PainFace

**Figure 2:**
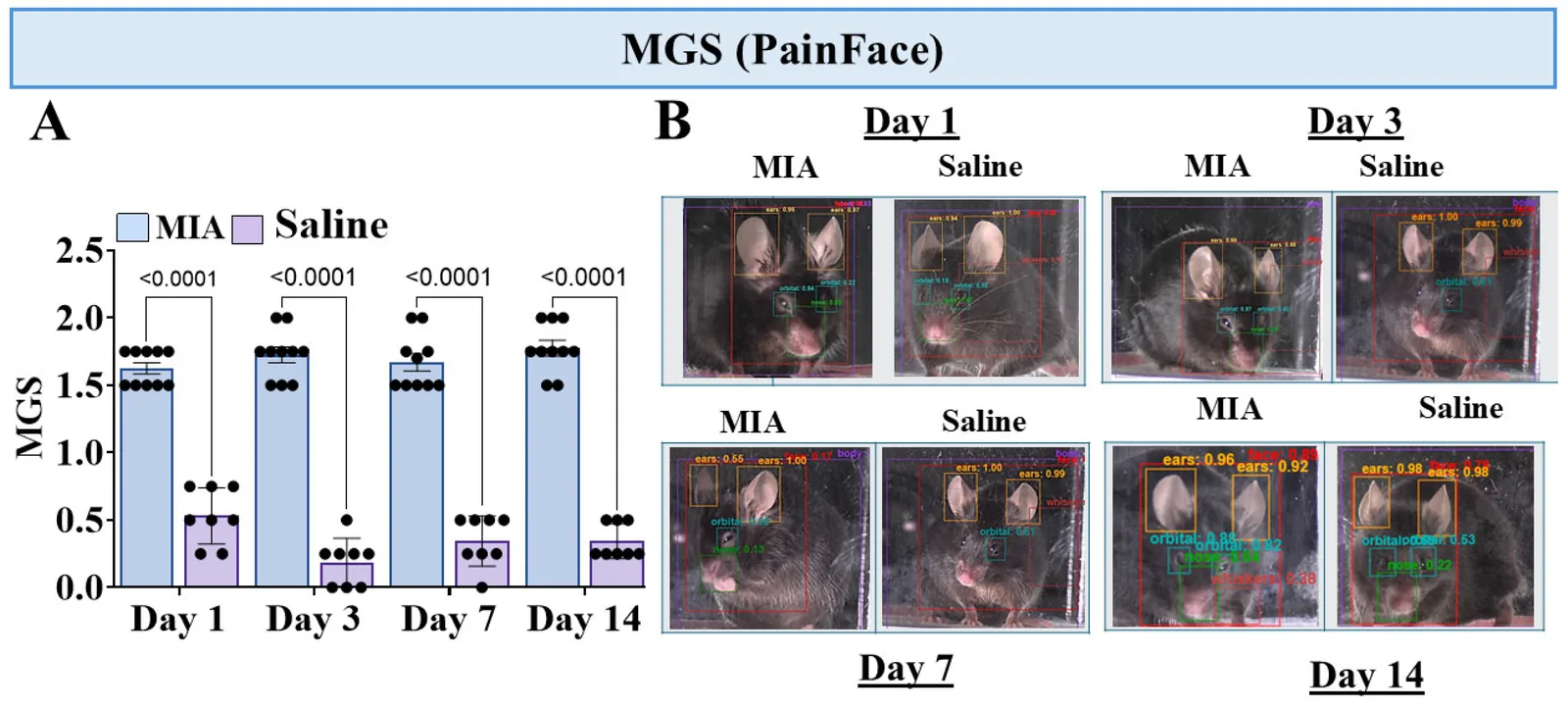
MIA induces pain-related spontaneous behaviors after intra-articular injection using the PainFace method. Representative facial images of MIA- and saline-treated mice on days 1, 3, 7, and 14 (**A**). Representative facial images of MGS scores on days 1, 3, 7, and 14 (**B**). Data are presented as mean ± SEM. *n* = 8–10 mice per group.

Pain-related spontaneous behaviors induced by MGS were assessed using PainFace. After intra-articular injection of MIA or saline, we assessed pain-related spontaneous behaviors on days 1, 3, 7, and 14 using the MGS. PainFace scoring revealed significantly higher facial pain scores in MIA-treated mice compared with saline-treated mice on days 1, 3, 7, and 14 (Figure 2A and Two-Way ANOVA). Representative facial images showed distinct pain-associated facial expressions in MIA-treated mice compared with saline-treated controls on days 1, 3, 7, and 14 (Figure 2B).

## 4. Discussion

This study reveals three important findings. (1) Mice with OA induced by MIA develop pain-related spontaneous behavior, as measured by the MGS test. (2) Pain-related spontaneous behavior persists for at least 14 days after MIA administration. (3) Support is provided for the utility of MGS as a behavioral measure of pain-related facial expressions in the MIA model.

Pain is a significant public health issue affecting millions of individuals. It is defined as an unpleasant sensory and emotional experience associated with, or resembling that associated with, actual or potential tissue damage [13].

In people with OA, pain is often chronic and can persist for prolonged periods, substantially contributing to disability and reduced quality of life [7,14]. Patients with OA may also experience ongoing pain at rest, particularly as the disease progresses, in addition to pain triggered by joint movement or loading [15,16].

MGS was developed to quantify pain-related spontaneous behavior in rodents and has been shown to provide a reliable measure of pain across multiple experimental conditions [17,18]. Although the MGS is used to study ongoing pain in rodents, it has not been tested in MIA or any OA models. Here, we test the potential usefulness of this test in OA for studying pain-related spontaneous behavior.

Currently, there are two ways to assess MGS in rodents. Manual scoring is based on the investigator’s observation of facial expressions, and an automated approach uses PainFace software. Because this is the first study of MGS in any OA model, we used both manual and PainFace scoring to reliably assess MGS in MIA.

Intra-articular MIA administration produced a significant and sustained increase in MGS scores compared with saline-treated controls, with elevated scores observed on days 1, 3, 7, and 14 after OA induction. The persistence of increased MGS scores across these time points indicates that MIA-induced OA is associated with ongoing pain-related facial expressions rather than a transient response limited to the early post-injection period.

More recently, the PainFace platform used in the present study was developed to standardize and scale up mouse grimace analysis and was validated across independent laboratories, supporting its use for quantifying non-evoked pain-related spontaneous behavior [19,20]. Our data showed that the PainFace approach reproduced the high MGS, indicating ongoing pain and validating the manual scoring data. Therefore, our data support using MGS as a behavioral endpoint to investigate OA-associated ongoing pain.

We conducted longitudinal studies of ongoing pain following MIA injection. This is important because the persistence of ongoing pain is observed in humans with OA [7,14,21]. In the present study, we observed pain-related spontaneous behavior on days 1, 3, 7, and 14 after OA induction, demonstrating that OA induction produces sustained spontaneous pain.

In conclusion, the present study demonstrates that intra-articular MIA injections induce pain-related spontaneous behavior, and MGS is a valid and reliable test for studying pain-related spontaneous behavior in the MIA model of OA.

## Data Availability

The data that support the findings of this study are available on request from the corresponding author.
